# Tinnitus at the Junction of Traditional Medicine and Modern Technology

**DOI:** 10.3390/nu15081898

**Published:** 2023-04-14

**Authors:** Birgit Mazurek, Holger Schulze, Winfried Schlee, Christian Dobel

**Affiliations:** 1 Tinnitus Center, Charité—Universitätsmedizin Berlin, 10117 Berlin, Germany; 2 Department of Otorhinolaryngology—Head and Neck Surgery, Universitätsklinikum Erlangen, 91054 Erlangen, Germany; 3 Department of Psychiatry, Universitätsklinikum Regensburg, 93053 Regensburg, Germany; 4 Institute for Information and Process Management, Eastern Switzerland University of Applied Sciences, 9001 St. Gallen, Switzerland; 5 Department of Otorhinolaryngology, Jena University Hospital, 07743 Jena, Germany

## 1. Introduction

The WHO estimated that 430 million people worldwide suffer from moderate-to-severe hearing loss. This number will increase by 2050 to an estimate of around 700 million people who will need hearing rehabilitation [1]. Hearing loss is a common risk factor related to developing tinnitus, which involves the perception of a sound despite the absence of an external source [2]. Internationally, there is some agreement that the mere perception of a phantom sound has to be distinguished from the situation where the perception of the phantom sound is “associated with emotional distress, cognitive dysfunction, and/or autonomic arousal, leading to behavioural changes and functional disability” [3].

Tinnitus is a frequent symptom and affects a significant proportion of the general population. Although tinnitus prevalence rates vary considerably, European studies estimate a range between 11% and 30% and suggest an increased occurrence with age, hearing impairment, or male gender [4,5]. The prevalence of chronic diseases, including tinnitus, increased in the last 10 years, with more frequently occurring in relation to combinations of several chronic disorders, or combinations with chronic and acute disorders [6].

Tinnitus, as an example of a chronic disorder, also has a substantial economic impact, with estimated costs for our societies of approximately EUR 117–325 billion annually in the EU [7,8]. On a phenomenological level, emotional and cognitive difficulties can precede, exacerbate, or result from tinnitus, and studies point to depressive mood as a key factor in rendering tinnitus distress. Tinnitus, especially in severe cases, seems not to appear in an isolated manner, but it is highly comorbid [9]. Typical psychological, psychosomatic, and psychiatric comorbidities in tinnitus are affective disorder, anxiety disorders, somatoform disorders, and adjustment disorders, as well as impairments, which result from tinnitus and comorbidities. They impact patients’ lives on different levels: (a) the cognitive–emotional response systems (e.g., impaired concentration, loss of control), (b) impairment of behavioural response systems (e.g., difficulty falling asleep and staying asleep, social withdrawal), (c) communication disorder (e.g., hyperacusis), and (d) impairment of the physiological response system (e.g., myofascial imbalance in the cervical spine) [2,10].

Correspondingly, studies have highlighted the potentially severe effects of acute stress on auditory processing. These processes have also been implicated in mood and affective disorders, which involve changes in attention and cognition, accompanied by alterations in the HPA axis [11,12,13,14]. Stress, pain, fatigue, autonomy, and education level are multiple factors influencing the amount of perceived tinnitus distress [15]. In a linear regression model across various psychometric, audiological, and psychosocial influence parameters, depressive fatigue symptoms (concentration, sleep, rumination, decreased pleasure), experiencing emotional distress, somatization tendencies (pain experience, physician contacts), and age were significant predictors for tinnitus distress [15]. 

In a pioneering study on possible subgroups in tinnitus patients, four phenotypes were described [16]. Group 1 (avoiders) represents a large proportion of patients. Apart from the symptom tinnitus, patients reported few other symptoms. Group 2 (psychosomatic group) represents 15% of patients who had clinically relevant impairments in depression, anxiety, and perceived stress, in addition to high tinnitus distress. Patients in this subgroup report severely impaired quality of life and reduced coping skills with more pessimism, less experienced self-efficacy, and optimism. In this group, depressive or anxious symptoms are seen as a crucial basis for the overall symptom burden. This subgroup has a higher proportion of women and patients living alone, unemployed, and with lower overall educational status. A third subgroup (somatic group) is defined primarily by physical tension symptoms due to stress and/or underlying medical conditions. Here, for example, a strong correlation to pain experience is evident. The fourth subgroup (distress group) shows a strong correlation of tinnitus with above-average feelings of stress, accompanied by physical exhaustion and anxious–depressive mood. This group tends to include younger, working patients (more men) who report chronic stress and may be prone to burnout syndrome with subjectively diminished capacity, including mental capacity. 

On a neurophysiological level, research increasingly suggests that tinnitus is a multidimensional phenomenon, with auditory processing (sensory pathways) being involved, and suffering from tinnitus (limbic medial structures) is observed. Executive or attentional networks for control appear to be involved. In this regard, connections between frontal and limbic brain segments in tinnitus networks have been well studied and are associated with frequent affective comorbidity. The nuanced connections between the frontal cortex and limbic structures are well established, in addition to their role in clinical or subclinical affective states [17,18]. Functional connectivity studies, in particular, show clear involvement of the auditory cortex, association areas, cognitive processing (especially attention), and suppression in frontal areas, and emotional processing in the medial brain sections is also involved [3,17,18,19,20,21,22,23,24,25,26,27,28]. 

This situation necessarily requires a paradigm shift in approaching the problem: chronic comorbid diseases should be considered as states of a multifactorial and complex system (the human being), which is perturbed and pushed into a pathological, imbalanced state through the influence of multiple factors (genetic background, molecular parameters, psychological factors, and events, such as trauma or age-related physiological changes, which are modulated, for example, by a certain lifestyle, nutrition, hearing ability, smoking, behavioural habits, physical activity, sex, or gender).

This Special Issue of Nutrients aims to focus on a collection of basic research and clinical studies detailing advancements in the field of hearing loss and tinnitus, with a focus on vulnerability factors. The aim is to better understand the trajectory of tinnitus-related maladaptation, e.g., to identify novel biomarkers, as well as to find links/approaches that account for the complex phenotypes of comorbidity, including factors (e.g., nutrition, lifestyle, sex, and gender) in tinnitus and hearing loss.

Noise is one of the main causal and risk factors for tinnitus. Thus, studies from the basic sciences, targeting support medicine (e.g., nutrients and pharmacological interventions), are particularly relevant to understand the development and maintenance of tinnitus. The work by Tziridis and Schulze [29], for example, investigated possible effects of a Ginkgo extract on tinnitus development in their animal study. They demonstrate that noise trauma-induced cochlear synaptopathy, which has been shown to induce tinnitus, can be prevented if the extract is given before the trauma, but not if it is given after the trauma. In the preventive condition, Ginkgo-treated animals even showed increased cochlear innervation compared to placebo controls. The first studies conducted in humans suggest that dietary factors have an important role in the onset and severity of tinnitus. Jarach and colleagus [30] provide the first evidence for an inverse association between tinnitus onset and a diet characterized by protein-rich food and caffeine and highlight the importance of food variety: the variety of food decreased the risk of tinnitus (OR 0.47; 95% CI, 0.24–0.90). Marcrum and coauthors [31] show that the intake of caffeine, alcohol, and salt has a mild effect on tinnitus severity, but only in a small proportion of patients. Targeting blood parameters provided some insights into the generation and maintenance of tinnitus. Boecking and collegues report elevated levels of total cholesterol (non-HDL-c, LDL-d and lipoprotein-a) accompanied by high rates of being overweight and smoking [32]. Finally, in a system medicine approach, Mazurek and colleagues proposed an integrated approach, which spanned disorder, symptoms, and contributing factors [33]. 

## 2. The Problem: Comorbid, Chronic Diseases Are Diagnosed and Treated in Isolation, Hampering Efficient Treatment

The causal relationship between a specific factor (e.g., genetic mutation, infection by a virus, and injury) and health deterioration is not always applicable in the context of chronic diseases, and a long-lasting course, variance in pattern of strong symptom severity and frequency, as well as unclear pathophysiology are all observed. Identification of an isolated cause able to predict disease occurrence and course is extremely challenging, especially in the frequent case of comorbidity and when symptoms overlap and do not allow for easy separation of distinct diseases. This fact does not only affect knowledge about pathophysiology and its anatomical and functional layers, but it also significantly influences clinical assessment and its outcome: comorbid diseases are typically treated in isolation, where healthcare professionals tend to assign certain symptoms to the disease, which is more relevant to their discipline. Consequently, a treatment plan is often inefficient, especially in the presence of comorbidities, particularly in the early stages of the disease. Often, the comorbid diseases become chronic, turning a nuisance into a severely debilitating condition that renders normal life impossible and can persist for decades or a lifetime.

## 3. The Solution: Treating Comorbid Diseases as Symptoms of a Disturbed System and Develop Early Risk Predictors for Chronification

It can be assumed that the perception of ear noise as stressful has to do with deviant processing networks in the brain. Both a more peripheral onset of hearing loss with central consequences due to neural plasticity and altered limbic activity in the brain evoked by stress or depression may contribute. In any case, a multifactorial aetiology must be assumed for chronic tinnitus, whereby individual factors do not sufficiently explain the phenomenon as a whole and, for example, do not allow statements about therapeutic controllability.

We need a paradigm shift in approaching the problem: chronic comorbid diseases are states of a multifactorial and complex system (the human) that is perturbed and pushed into a pathological, imbalanced state through the influence of multiple factors (genetic background, as well as events such as trauma or age-related physiological changes, which are modulated, for example, by a certain lifestyle, smoking, behavioural habits, physical activity, sex, or gender). Therefore, the chronification of these diseases represents a state transition between a healthy individual and one that experiences a severe, debilitating long-term condition. This paradigm shift and possible solutions are discussed with a system medicine approach [33].

Tinnitus, depression, and chronic pain are ideal to establish this paradigm shift: they comprise mental and physical aspects (hence covering a broad spectrum of possible disease states), and they are highly prevalent and comorbid with each other and additional diseases, hence allowing one to disentangle common mechanisms and increasing the chance of finding generalisable principles [33]. Furthermore, they manifest in a broad spectrum of severities, ranging from negligible (at some point in their lives, almost everyone hears a beep in absence of a physical sound [34], feels depressed, or experiences back pain), to severely debilitating (hearing a constant unnerving tinnitus sound, being too depressed to live normally, or experiencing chronic pain that requires early retirement). The current literature shows that the likelihood of developing the conditions addressed is significantly linked also to certain unhealthy lifestyle choices or conditions, such as constant stress, or lack of physical exercise, to name a few. 

## 4. A Personalised Systems Medicine Approach for Tinnitus

**Figure 1:**
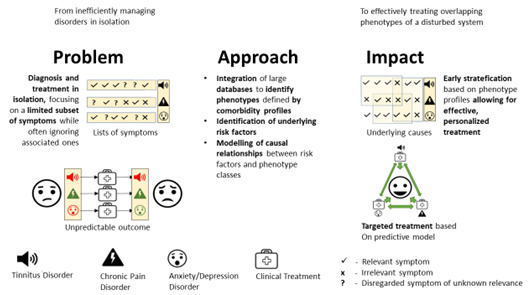
Personalised systems medicine approach.

For the future, we need new technological approaches (see Figure 1) for a more profound and precise understanding of the transients of pathological development. Identification of such pathways will, consequently, provide an opportunity for the identification of predictors (e.g., environmental factors, behavioural aspects, and biomarkers) of the development of diseases. The mere description of such predictors can result in strategies for the prevention and therapy of tinnitus or other complex psychosomatic clinical pictures. In the process, existing data sets can also be reprocessed with modern statistical methods to explore certain risk factors (e.g., nutritional characteristics) and their impact on the expression, chronification, or cure of tinnitus and its comorbidities.
